# A new karst-dwelling bent-toed gecko (Squamata: Gekkonidae: *Cyrtodactylus*) from Xiangkhoang Province, northeastern Laos

**DOI:** 10.24272/j.issn.2095-8137.2018.010

**Published:** 2018-04-28

**Authors:** Roman A. Nazarov, Olivier S.G. Pauwels, Evgeniy L. Konstantinov, Anatoliy S. Chulisov, Nikolai L. Orlov, Nikolay A. Poyarkov

**Affiliations:** 1 Zoological Museum, Moscow State University, Moscow 125009, Russia; 2 Royal Belgian Institute of Natural Sciences, Brussels B-1000, Belgium; 3 Institute of Natural Sciences, Kaluga State University named after K.I. Tsiolkovskii, Kaluga 248023, Russia; 4 Zoological Institute, Russian Academy of Sciences, St. Petersburg 199034, Russia; 5 Department of Vertebrate Zoology, Biological Faculty, Lomonosov Moscow State University, Moscow 119234, Russia; 6 Joint Russian-Vietnamese Tropical Research and Technological Center, 63 Nguyen Van Huyen Road, Nghia Do, Cau Giay, Hanoi, Vietnam

**Keywords:** Indochinese region, Karst, Limestone, Herpetology, Taxonomy, New species

## Abstract

We describe a new karst-dwelling *Cyrtodactylus* from Ban Thathom, Xiangkhoang Province, northeastern Laos. The new species can be distinguished from other congeners by having four dark dorsal bands between limb insertions, a discontinuous nuchal loop, 10 precloacal pores in males or 10–12 precloacal pits (females) separated by a diastema from a series of enlarged femoral scales bearing 18 or 19 pores (male) or 8–10 pits (females) along each femur, 14–18 dorsal tubercle rows at midbody, no precloacal groove, 30–36 midbody scale rows across belly between ventrolateral skin folds, transversely enlarged subcaudal plates, and a maximal known snout-vent length of 75.5 mm. Our description brings to 22 the number of *Cyrtodactylus* species recorded from Laos.

## 1. INTRODUCTION

The diversity of the gecko genus *Cyrtodactylus* has increased at a phenomenal rate. Of the more than 250 species recognized within the genus, about 80% of the diversity has been described in the twenty-first century (Uetz et al., 2018). Numbers of *Cyrtodactylus* species in Thailand and Vietnam have shown considerable expansion, including at least 33 and 39 recognized species in each country, respectively (Pauwels et al., 2016). Moreover, several species remain to be named in Vietnam, in particular within the *Cyrtodactylus irregularis* species complex (Nguyen et al., 2017). Although Laos is located between these two species-rich countries and harbors a varied geological relief propitious to micro-endemism, its *Cyrtodactylus* fauna is currently known to include only 21 species (Luu et al., 2016a; Nazarov et al., 2014). Such low species number may be due to the fewer herpetological surveys conducted in Laos compared to Vietnam and Thailand, particularly on the karst reliefs (Teynié & David, 2014).

Our team co-described five of the *Cyrtodactylus* species currently recorded from Laos (Nazarov et al., 2014; Ngo & Pauwels, 2010), and we are pursuing our efforts to better inventory the diversity of the genus in this country.

During fieldwork in the forested karst massifs in the Laotian province of Xiangkhoang, we (A.S.C. and E.L.K.) encountered three geckos that differed in color pattern and scalation from known species, and which we show hereafter to belong to an undescribed species. Morphological characters, such as scalation and dorsal color and pattern, indicate that this newly discovered *Cyrtodactylus* population clearly belongs to the *C. phongnhakebangensis* species group *sensu* Luu et al. (2016a), which includes a dozen species from Laos and Vietnam. In agreement with the definition of this group provided by Luu et al. (2016a: 132), this new species exhibits a maximum adult SVL comprised between 73.0 and 100.6 mm, 0 or 1 supranasal, a DorTub (14–18) between 10 and 24, no webbing between fingers or toes, tubercles nearly absent on forelimbs but present on hind limbs, precloacal and femoral pores in males (in total 47) between 20 and 60, number of postcloacal tubercles (5 or 6 in male) between 3 and 8, enlarged subcaudals, and dorsum displaying well-defined dark bands.

## MATERIALS AND METHODS

### Sampling

Field surveys were conducted in Xiangkhoang Province, Laos, from May to July 2008 within the frameworks of the Memorandum of Understanding (MOU) on the Academic and Scientific Cooperation between the National University of Laos and Kaluga State University, Russia (MOU No. 72 20-11-2012). Specimens were collected by hand from 1900 h to 2300 h. Specimens were photographed in life and subsequently euthanized in a closed vessel with a piece of cotton wool containing ethyl acetate (Simmons, 2002). Tissue samples were preserved separately in 95% ethanol and specimens were fixed in 85% ethanol, after which they were transferred to 70% ethanol for permanent storage. Specimens were subsequently deposited in the herpetological collection of the Zoological Institute of the Russian Academy of Science (ZISP) and in the herpetological collection of the Zoological Museum of Lomonosov Moscow State University (ZMMU), Moscow, Russia.

**Figure 1:**
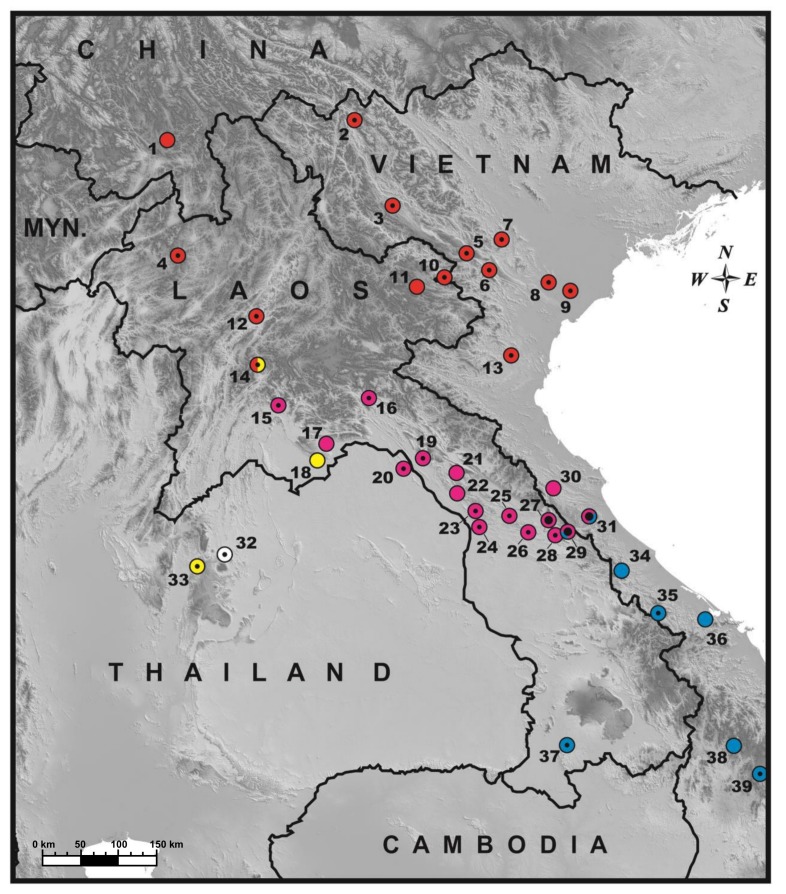
General distribution of the genus *Cyrtodactylus* in Laos and surrounding areas

The specimens accessed for molecular analyses are listed in Table 1. Our sampling is to date the most comprehensive sampling covering all known lineages of the *C. phongnhakebangensis* species complex. A map showing the distribution of the *C. phongnhakebangensis* species complex in Laos and adjacent territories and the location of the sampling site for the present work is provided in Figure 1. Museum abbreviations used are listed as follows: CAS: California Academy of Sciences, San Francisco; CUMZ R: Chulalongkorn University Museum of Zoology (Reptiles), Bangkok; IRSNB: Institut Royal des Sciences Naturelles de Belgique, Brussels; KZM: Khorat Zoo Museum, Nakhon Ratchasima; MNHN: Muséum National d’Histoire Naturelle, Paris; PSUZC-RT: Prince of Songkhla University Zoological Collection, Reptile Section, Songkhla; QSMI: Queen Saovabha Memorial Institute, Thai Red Cross Society, Bangkok; THNHM: Thailand Natural History Museum, National Science Museum, Technopolis, Pathum Thani; ZMMU: Zoological Museum, Moscow State University, Moscow; ZISP: Zoological Institute, Saint Petersburg. Comparisons with congeneric species are based on literature data and on direct examination of preserved specimens (Appendix I).

**Figure 2:**
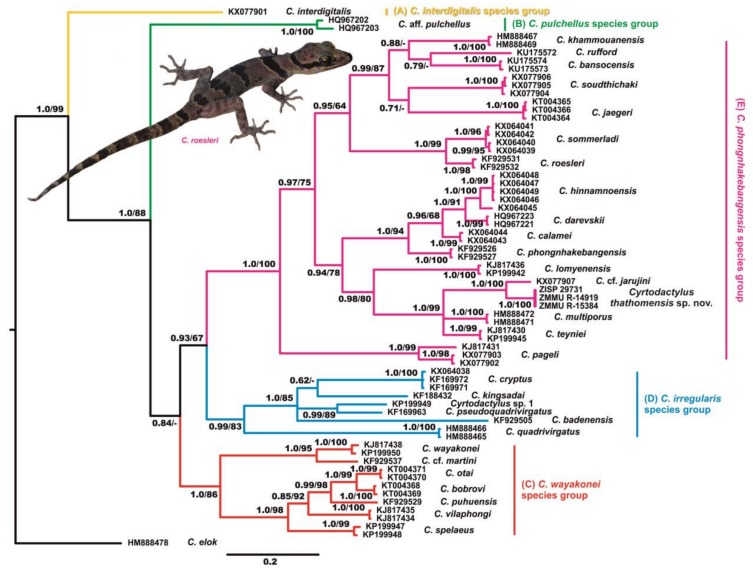
Bayesian inference (BI) tree for the *Cyrtodactylus phongnhakebangensis* species group and other *Cyrtodactylus* species groups inhabiting Laos and adjacent areas based on analysis of *COI* gene fragments

For detailed locality information see Appendix II. Icon colors correspond to the species groups as shown in Figure 2: A: Yellow, *C. interdigitalis* species group; B: Green, *C. pulchellus* species group; C: Red, *C. wayakonei* species group; D: Blue, *C. irregularis* species group; E: Purple, *C. phongnhakebangensis* species group; dot in icon center indicates type locality; large dot indicates type localities for several species of *Cyrtodactylus*. MYN—Myanmar.

Clade colors correspond to icon colors in Figure 1. Values at nodes correspond to BI PP/ML BS support values, respectively. Sequence of *C. elok* was used as an outgroup. Inset shows *C. roesleri*, a representative of the *C. phongnhakebangensis* species group.

**Table 1:** Specimens and sequences of *Cyrtodactylus* representatives used in molecular analyses of mtDNA gene fragment

No.	Species	GenBank accession No.	Locality	Voucher ID
1	*Cyrtodactylus badenensis*	KF929505	Vietnam, Tay Ninh Prov.	KIZ13689
2	*C. bansocensis*	KU175573	Laos, Khammouane Prov.	VNUF–R.2015.20
3	*C. bansocensis*	KU175574	Laos, Khammouane Prov.	NUOL-R-2015.21
4	*C. bobrovi*	KT004368	Vietnam, Hoa Binh Prov.	IEBR-A.2015.30
5	*C. bobrovi*	KT004369	Vietnam, Hoa Binh Prov.	VNMN-A.2015.61
6	*C. calamei*	KX064043	Laos, Khammouane Prov.	NUOL-R-2015.22
7	*C. calamei*	KX064044	Laos, Khammouane Prov.	VNUF-R.2015.28
8	*C. cryptus*	KF169971	Vietnam, Quang Binh Prov.	PNKB3
9	*C. cryptus*	KF169972	Vietnam, Quang Binh Prov.	PNKB4
10	*C. cryptus*	KX064038	Laos, Khammouane Prov.	VNUF-R.2014.69
11	*C. elok*	HM888478	Malaysia	ZMMU-RAN1991
12	*C. darevskii*	HQ967221	Laos, Khammouane Prov.	ZISP-FN256
13	*C. darevskii*	HQ967223	Laos, Khammouane Prov.	ZISP-FN223
14	*C. hinnamnoensis*	KX064045	Laos, Khammouane Prov.	IEBR-A.2013.89
15	*C. hinnamnoensis*	KX064046	Laos, Khammouane Prov.	IEBR-A.2013.90
16	*C. hinnamnoensis*	KX064047	Laos, Khammouane Prov.	VNUF-R.2015.11
17	*C. hinnamnoensis*	KX064048	Laos, Khammouane Prov.	VNUF-R.2015.3
18	*C. hinnamnoensis*	KX064049	Laos, Khammouane Prov.	NUOL-R-2015.9
19	*C. lomyenensis*	KJ817436	Laos, Khammouane Prov.	IEBR-KM2012.54
20	*C. lomyenensis*	KP199942	Laos, Khammouane Prov.	IEBR-KM2012.52
21	*C. interdigitalis*	KX077901	Laos, Khammouane Prov.	VNUF-R.2014.50
22	*C. jaegeri*	KT004364	Laos, Khammouane Prov.	IEBR-A.2013.55
23	*C. jaegeri*	KT004365	Laos, Khammouane Prov.	NUOL-R.2013.1
24	*C. jaegeri*	KT004366	Laos, Khammouane Prov.	VFU-TK914
25	*C.*cf. *jarujini*	KX077907	Laos, Bolikhamxay Prov.	VNUF-R.2015.7
26	*C. khammouanensis*	HM888467	Laos, Khammouane Prov.	ZISP-FN191
27	*C. khammouanensis*	HM888469	Laos, Khammouane Prov.	ZISP-FN257
28	*C. kingsadai*	KF188432	Vietnam, Phu Yen Prov.	IEBR-A.2013.3
29	*C.*cf. *martini*	KF929537	China, Yunnan Prov.	KIZ201103
30	*C. multiporus*	HM888472	Laos, Khammouane Prov.	ZISP-FN3
31	*C. multiporus*	HM888471	Laos, Khammouane Prov.	ZISP-FN2
32	*C. otai*	KT004370	Vietnam, Hoa Binh Prov.	IEBR-A.2015.26
33	*C. otai*	KT004371	Vietnam, Hoa Binh Prov.	IEBR-A.2015.27
34	*C. puhuensis*	KF929529	Vietnam, Thanh Hoa Prov.	KIZ11665
35	*C.*aff. *pulchellus*	HQ967202	Malaysia	ZMMU-R-12643-3
36	*C.*aff. *pulchellus*	HQ967203	Malaysia	ZMMU-R-12643-4
37	*C. pageli*	KJ817431	Laos, Vientiane Prov.	ZFMK91827
38	*C. pageli*	KX077902	Laos, Vientiane Prov.	NQT2010.36
39	*C. pageli*	KX077903	Laos, Vientiane Prov.	NQT2010.37
40	*C. phongnhakebangensis*	KF929526	Vietnam, Quang Binh Prov.	PNKB2011.30
41	*C. phongnhakebangensis*	KF929527	Vietnam, Quang Binh Prov.	PNKB2011.32
42	*C. pseudoquadrivirgatus*	KF169963	Vietnam, Thua Thien - Hue Prov.	ITBCZ3001
43	*Cyrtodactylus*sp. 1	KP199949	Vietnam, Da Nang, Ba Na	ZMMU-R-13095-2
44	*C. quadrivirgatus*	HM888465	Malaysia	ZMMU-RAN1989
45	*C. quadrivirgatus*	HM888466	Malaysia	ZMMU-RAN1990
46	*C. roesleri*	KF929532	Vietnam, Quang Binh Prov.	PNKB2011.34
47	*C. roesleri*	KF929531	Vietnam, Quang Binh Prov.	PNKB2011.3
48	*C. rufford*	KU175572	Laos, Khammouane Prov.	VFU-R.2015.14
49	*C. sommerladi*	KX064039	Laos, Khammouane Prov.	IEBR-A.2015.37
50	*C. sommerladi*	KX064040	Laos, Khammouane Prov.	VNUF-R.2013.22
51	*C. sommerladi*	KX064041	Laos, Khammouane Prov.	VNUF-R.2013.87
52	*C. sommerladi*	KX064042	Laos, Khammouane Prov.	IEBR-A.2015.39
53	*C. spelaeus*	KP199947	Laos, Vientiane Prov.	ZMMU-R-13980-3
54	*C. spelaeus*	KP199948	Laos, Vientiane Prov.	ZMMU-R-13980-1
55	*C. soudthichaki*	KX077904	Laos, Khammouane Prov.	NUOL-R-2015.5
56	*C. soudthichaki*	KX077905	Laos, Khammouane Prov.	VFU-R.2015.18
57	*C. soudthichaki*	KX077906	Laos, Khammouane Prov.	IEBR-A.2015.34
58	*C. teyniei*	KJ817430	Laos, Khammouane Prov.	IEBR-KM2012.77
59	*C. teyniei*	KP199945	Laos, Khammouane Prov.	IEBR-KM2012.77
60	*C. vilaphongi*	KJ817434	Laos, Luang Prabang Prov.	NUOL-R-2013.5
61	*C. vilaphongi*	KJ817435	Laos, Luang Prabang Prov.	IEBR-A.2013.103
62	*C. wayakonei*	KJ817438	Laos, Luang Nam Tha Prov.	ZFMK91016
63	*C. wayakonei*	KP199950	Laos, Luang Nam Tha Prov.	ZMMU-R-13981-1
64	*Cyrtodactylus thathomensis* **sp. nov.**	MG791873	Laos, Xiangkhouang Prov.	ZMMU-R-14919-1
65	*Cyrtodactylus thathomensis* **sp. nov.**	MG791874	Laos, Xiangkhouang Prov.	ZISP 29731
66	*Cyrtodactylus thathomensis* **sp. nov.**	MG791875	Laos, Xiangkhouang Prov.	ZMMU R-15384

### Morphological descriptions

Measurements and meristic counts follow Pauwels et al. (2016). Measurements were taken on the right side. Paired meristic characters are given in left/right order. Numbers of supralabial and infralabial scales were counted from the largest scale immediately posterior to the dorsal inflection of the posterior portion of the upper jaw to the rostral and mental scales, respectively; the number of longitudinal rows of body tubercles was counted transversely across the center of the dorsum from one ventrolateral skin fold to the other; the number of longitudinal rows of ventral scales was counted transversely across the center of the abdomen from one ventrolateral skin fold to the other; subdigital lamellae beneath the toes were counted from the base of the first phalanx to the claw; dorsal dark bands between limb insertions are those strictly contained on the dorsum between the posterior insertion of the anterior limbs and the anterior insertion of the posterior limbs. In previous literature, dorsal bands often include those contained between the anterior insertion point of the anterior limbs and the posterior point of insertion of the posterior limbs, thus interspecific comparisons on band numbers were performed with caution.

The following measurements were taken with a digital caliper to the nearest 0.1 mm: AG: axilla to groin length, taken from the posterior margin of the forelimb at its insertion point on the body to the anterior margin of the hind limb at its insertion point on the body; EarL: ear length, the greatest horizontal distance of the ear opening; ForeaL: forearm length, taken on the dorsal surface from the posterior margin of the elbow while flexed 90${}^{\circ }$ to the inflection of the flexed wrist; HeadH: head height, the maximum height of head from the occiput to the throat; HeadL: head length, from the posterior margin of the retroarticular process of the lower jaw to the tip of the snout; HeadW: head width, measured at the angle of the jaws; Internar: internarial distance, measured between the nares across the rostrum; Interorb: interorbital distance, measured between the anterior edges of the orbits; ML: mental length, the maximum length of mental shield; MW: mental width, the maximum width of mental shield; NosOrb: nostril to orbit distance, from the posterior margin of the external nares to the anterior margin of the orbit; OrbD: orbit diameter, the greatest horizontal diameter of the orbit; OrbEar: orbit to ear distance, from the anterior edge of the ear opening to the posterior edge of the orbit; RH: rostral height, the maximum height of the rostral shield; RW: rostral width, the distance between border of rostral shield and the first supralabial scales on right and left sides; SnOrb: snout to orbit distance, from the tip of the snout to the anteriormost margin of the orbit; SVL: snout-vent length, taken from the tip of snout to the vent; TailL: tail length, taken from the vent to the tip of the tail, original or regenerated; TailW: tail width, taken at the base of the tail immediately posterior to the postcloacal swelling; TibiaL: tibia length, taken on the ventral surface from the posterior surface of the knee while flexed 90${}^{\circ }$ to the base of heel. Meristic characters abbreviations: DorTub: number of longitudinal rows of dorsal tubercles at midbody; EnlFemSc: enlarged femoral scales; FemPi: femoral pits; FemPo: femoral pores; FemPreclPo: number of femoral and precloacal pores in continuous series; IL: infralabial scales; InterorbSc: interorbital scales; ParaTub: number of paravertebral tubercles between the limbs insertions, counted in a straight line immediately left of the vertebral column; PreclPi: precloacal pits (shallow depressions without waxy exudates); PreclPo: precloacal pores (deeper than pits, and with waxy exudates); SL: supralabial scales; SLF4: subdigital lamellae beneath 4^th^ finger (basal and distal lamellae combined); SLT4: subdigital lamellae beneath 4^th^ toe (basal and distal lamellae combined); Ven: number of ventral scale rows.

### DNA isolation, PCR, and sequencing

For molecular phylogenetic analyses, total genomic DNA was extracted from ethanol-preserved femoral muscle tissue (Table 1) using standard phenol-chloroform-proteinase K (final concentration 1 mg/mL) extraction procedures with consequent isopropanol precipitation (protocols followed Hillis et al., 1996 and Sambrook et al., 1989). The isolated total genomic DNA was visualized using agarose electrophoresis in the presence of ethidium bromide. The concentration of total DNA was measured in 1 μL using NanoDrop 2000 (Thermo Scientific, USA), and consequently adjusted to ca. 100 ng DNA/μL.

The mitochondrial cytochrome oxidase subunit I (*COI*) was selected as a genetic marker to clarify the taxonomic position of the newly discovered population of *Cyrtodactylus*. A total of 655 bp of *COI* was amplified using a mitochondrial marker widely used as a barcoding marker for vertebrates, including both reptiles and amphibians (Murphy et al., 2013; Nagy et al., 2012; Smith et al., 2008), and which proved to be useful for species identification and assessment of cryptic diversity in various groups of lizards, including the genus *Cyrtodactylus* (Brennan et al., 2017; Hartmann et al., 2013; Luu et al., 2016a; Nazarov et al., 2012, 2014; Nguyen et al., 2013, 2014, 2017; Solovyeva et al., 2011). Primers used both for PCR and sequencing were the VF1-d (5′-TTCTCAACCAACCACAARGAYATYGG-3′) and the VR1-d (5′-TAGACTTCTGGGTGGCCRAARAAYCA-3’) (following Ivanova et al., 2006). The obtained fragments were sequenced in both directions for each sample, and a consensus sequence was generated. PCR analyses were performed in 25 μL reactions using ca. 50 ng genomic DNA, 10 pmol of each primer, 15 nmol of each dNTP, 50 nmol additional MgCl_2_, Taq PCR buffer (10 mmol/L Tris-HCl, pH 8.3, 50 mmol/L KCl, 1.1 mmol/L MgCl_2_, and 0.01% gelatin), and 1 U of Taq DNA polymerase. The PCR conditions for the *COI* gene fragment followed Nazarov et al. (2012) and included an initial denaturation step at 95 ${}^{\circ }$C for 3 min; 5 cycles at 95 ${}^{\circ }$C for 30 s, annealing at 45 ${}^{\circ }$C for 1 min, extension at 72 ${}^{\circ }$C for 2 min, followed with 35 cycles at 95 ${}^{\circ }$C for 30 s, annealing at 51 ${}^{\circ }$C for 1 min, extension at 72 ${}^{\circ }$C for 2 min, and a final extension at 72 ${}^{\circ }$C for 5 min.

The PCR products were loaded onto 1.5% agarose gels in the presence of ethidium bromide and visualized using agarose electrophoresis. If distinct bands were produced, products were purified using 2 μL, from a 1:4 dilution of ExoSapIt (Amersham, UK), per 5 μL of PCR product prior to cycle sequencing. A 10 μL sequencing reaction included 2 μL of template, 2.5 μL of sequencing buffer, 0.8 μL of 10 pmol primer, 0.4 μL of BigDye Terminator version 3.1 Sequencing Standard (Applied Biosystems), and 4.2 μL of water. The cycle sequencing reaction included 35 cycles of 10 s at 96 ${}^{\circ }$C, 10 s at 50${}^{\circ }$ C, and 4 min at 60 ${}^{\circ }$C. Cycle sequencing products were purified by ethanol precipitation. Sequence data collection and visualization were performed on an ABI 3730xl automated sequencer (Applied Biosystems, USA). The obtained sequences were deposited in GenBank under the accession numbers MG791873–MG791875 (Table 1).

### Phylogenetic analyses

A total of 66 *COI* sequences of congeners were obtained from GenBank for phylogenetic analyses, including the three sequences obtained in this study (Table 1).

Nucleotide sequences were initially aligned using ClustalX 1.81 (Thompson et al., 1997) with default parameters, and then optimized manually in BioEdit 7.0.5.2 (Hall, 1999) and MEGA 6.0 (Tamura et al., 2013). Mean uncorrected genetic distances (*P*-distances) between sequences were determined with MEGA 6.0. MODELTEST v.3.06 (Posada & Crandall, 1998) was used to estimate the optimal models of DNA evolution. The best-fitting model selected for *COI* dataset was K80+I+G for the first and the second codon positions and GTR+G for the third codon position, as suggested by the Akaike Information Criterion (AIC).

Phylogenetic trees were inferred using two different methods: Bayesian inference (BI) and maximum likelihood (ML). BI was conducted in MrBayes 3.1.2 (Huelsenbeck & Ronquist, 2001; Ronquist & Huelsenbeck, 2003). Metropolis-coupled Markov chain Monte Carlo (MCMCMC) analyses were run with one cold chain and three heated chains for four million generations and sampled every 1 000 generations. Five independent MCMCMC runs were performed and 1 000 trees were discarded as burn-in. Confidence in tree topology was assessed by posterior probability (BI PP) (Huelsenbeck & Ronquist, 2001). The ML analyses were conducted using Treefinder (Jobb et al., 2004). The *COI* sequence of *Cyrtodactylus elok* Dring was used as an outgroup following Luu et al. (2016a). Confidence in tree topology was tested by non-parametric bootstrap analysis (ML BS) with 1 000 replicates (Felsenstein, 2004). We *a priori* regarded tree nodes with bootstrap (ML BS) values of 70% or greater and posterior probabilities (BI PP) values over 0.95 as sufficiently resolved, ML BS values between 70% and 50% (and BI PP between 0.95 and 0.90) were regarded as tendencies, and ML BS values below 50% (BI PP below 0.90) were considered unresolved (Felsenstein, 2004; Huelsenbeck & Hillis, 1993).

## RESULTS

### Genetic differentiation

#### Sequence data

The final alignment of the examined mtDNA *COI* gene fragment consisted of 672 sites: 386 sites were conserved and 286 sites were variable, of which 270 were found to be potentially parsimony-informative. The transition-transversion bias (R) was estimated as 3.66. Nucleotide frequencies were A=23.14%, T=26.77%, C=30.53%, and G=19.95%.

### mtDNA genealogy

Results of the phylogenetic analyses implemented in the present paper are shown in Figure 2. The BI and ML analyses resulted in essentially similar topologies slightly differing from each other only in associations at several poorly supported basal nodes. In general, the genetic differentiation of the *Cyrtodactylus phongnhakebangensis* species group members was highly consistent with the results reported in previous studies by Nazarov et al. (2014) and Luu et al. (2016a). The partial fragment of the *COI* gene strongly supports monophyly of the *C. phongnhakebangensis* (1.0/100, hereafter node support values are given for BI PP/ML BS, respectively), *C. wayakonei* (1.0/86), and *C. irregularis* (0.99/83) species groups. Within the *C. phongnhakebangensis* species group, *C. pageli* Schneider, Nguyen, Schmitz, Kingsada, Auer & Ziegler, 2011 formed a sister lineage with respect to all other members of the *C. phongnhakebangensis* species group (0.97/75). The remaining members of the *C. phongnhakebangensis* species group were clustered in two subclades with moderate to strong support values: (1) subclade consisting of *C. khammouanensis* Nazarov, Poyarkov, Orlov, Nguyen, Milto, Martynov, Konstantinov & Chulisov, 2014, *C. rufford* Luu, Calame, Nguyen, Le, Bonkowski & Ziegler, 2016, *C. bansocensis* Luu, Nguyen, Le, Bonkowski & Ziegler, 2016, *C. soudthichaki* Luu, Calame, Nguyen, Bonkowski & Ziegler, 2015, *C. jaegeri* Luu, Calame, Bonkowski, Nguyen & Ziegler, 2014, *C. sommerladi* Luu, Bonkowski, Nguyen, Le, Schneider, Ngo & Ziegler, 2016, and *C. roesleri* Ziegler, Nazarov, Orlov, Nguyen, Vu, Dang, Dinh & Schmitz, 2010 (0.95/64); and (2) subclade consisting of *C. hinnamnoensis* Luu, Bonkowski, Nguyen, Le, Schneider, Ngo & Ziegler, 2016, *C. darevskii* Nazarov, Poyarkov, Orlov, Nguyen, Milto, Martynov, Konstantinov & Chulisov, 2014, *C. calamei* Luu, Bonkowski, Nguyen, Le, Schneider, Ngo & Ziegler, 2016, *C. phongnhakebangensis* Ziegler, Rösler, Herrmann & Vu, 2002, *C. lomyenensis* Ngo & Pauwels, 2010, *C. multiporus* Nazarov, Poyarkov, Orlov, Nguyen, Milto, Martynov, Konstantinov & Chulisov, 2014, *C. teyniei* David, Nguyen, Schneider & Ziegler, 2011, *C*. cf. *jarujini* Ulber, 1993, and *Cyrtodactylus* sp. from Ban Thathom, Xiangkhoang Province, Laos (0.94/78).

The newly discovered *Cyrtodactylus* population from Ban Thathom, Xiangkhoang Province, was placed within the first subclade of the *C. phongnhakebangensis* species group and was clustered in one group with *C. multiporus*, *C. teyniei*, and *C*. cf. *jarujini* (1.0/99). Among these species, the Ban Thathom population appeared to be a sister lineage of *C*. cf. *jarujini* from the Bolikhamxay Province of Laos (1.0/100).

### Genetic distances

The uncorrected genetic *P*-distances among and within the *COI* gene fragment of the studied members of the *Cyrtodactylus phongnhakebangensis* species group are summarized in Table 2. The observed interspecific distances in the *COI* gene within the *C. phongnhakebangensis* species group varied from *P*=4.2% (between *C. darevskii* and *C. hinnamnoensis*) to *P*=19.4% (between *C. soudthichaki* and *C. hinnamnoensis*) (Table 2). These values slightly overlapped with interspecific comparisons between the *C. phongnhakebangensis* species group members and the outgroup *Cyrtodactylus* species (18.1%<*P*<24.4%, data not shown in Table 2). The observed intraspecific distances in our analysis varied from *P*=0% to *P*=3.8%, with the last value corresponding to the genetic differentiation between mtDNA lineages of *C. pageli* (Table 2).

**Table 2:** Uncorrected *P*-distances (percentages) between *COI* sequences of the *Cyrtodactylus phongnhakebangensis* species group members included in phylogenetic analyses (below the diagonal), and standard error estimates (above the diagonal)

No.	Species	1	2	3	4	5	6	7	8	9	10	11	12	13	14	15	16	17
**1**	*C. thathomensis* **sp. nov.**	**0.0**	1.8	1.7	1.7	1.7	1.8	1.5	1.5	1.4	1.5	1.8	1.7	1.4	1.0	1.3	1.2	1.8
**2**	*C. khammouanensis*	18.3	**0.2**	1.4	1.4	1.4	1.5	1.4	1.3	1.4	1.5	1.3	1.4	1.4	1.8	1.6	1.5	1.6
**3**	*C. rufford*	18.9	11.5	**—**	1.3	1.6	1.5	1.6	1.5	1.6	1.5	1.5	1.5	1.5	1.8	1.4	1.5	1.7
**4**	*C. bansocensis*	17.1	12.1	11.2	**0.2**	1.6	1.5	1.4	1.3	1.7	1.8	1.5	1.6	1.4	1.6	1.4	1.6	1.6
**5**	*C. soudthichaki*	18.4	13.3	14.5	12.2	**0.1**	1.6	1.3	1.4	1.8	1.8	1.6	1.7	1.8	1.6	1.5	1.4	1.4
**6**	*C. jaegeri*	18.5	14.6	14.5	14.8	13.5	**0.1**	1.6	1.5	1.9	1.8	1.8	1.7	1.4	1.8	1.7	1.8	1.7
**7**	*C. sommerladi*	17.7	16.7	17.9	16.0	15.5	16.3	**0.1**	0.8	1.5	1.6	1.4	1.4	1.6	1.5	1.5	1.7	1.4
**8**	*C. roesleri*	17.9	16.5	16.4	15.9	15.2	15.4	6.0	**0.2**	1.6	1.7	1.4	1.4	1.5	1.4	1.4	1.6	1.3
**9**	*C. hinnamnoensis*	16.4	16.6	18.7	17.5	19.4	18.1	16.3	17.1	**0.9**	0.8	0.9	1.2	1.5	1.4	1.5	1.4	1.6
**10**	*C. darevskii*	16.2	14.8	18.5	16.7	18.5	17.8	15.7	17.3	4.2	**0.0**	1.0	1.4	1.6	1.6	1.8	1.6	1.6
**11**	*C. calamei*	17.3	13.7	15.6	16.4	17.6	17.3	15.2	15.5	5.2	5.6	**0.0**	1.1	1.4	1.8	1.6	1.6	1.5
**12**	*C. phongnhakebangensis*	16.0	15.5	17.6	15.4	17.7	16.7	16.3	15.3	9.1	9.6	7.8	**0.0**	1.3	1.6	1.7	1.8	1.4
**13**	*C. lomyenensis*	15.5	15.6	17.4	16.0	18.8	17.1	17.8	17.5	15.4	14.5	15.0	14.6	**0.2**	1.4	1.4	1.3	1.6
**14**	*C.*cf. *jarujini*	4.5	16.1	17.3	18.0	19.0	18.8	18.1	17.4	16.5	16.7	17.1	17.1	15.2	**—**	1.2	1.1	1.6
**15**	*C. multiporus*	9.5	16.6	15.8	16.4	18.3	16.1	17.0	17.0	15.4	15.6	15.5	15.3	14.4	9.3	**0.0**	1.0	1.5
**16**	*C. teyniei*	9.3	17.2	17.3	18.3	17.4	18.1	17.7	17.5	15.3	15.6	14.2	15.3	14.5	9.3	6.0	**0.0**	1.5
**17**	*C. pageli*	18.5	18.3	17.8	18.8	18.8	18.0	16.2	16.6	18.8	18.2	16.9	17.8	17.7	18.0	16.5	17.5	**3.8**

Ingroup mean uncorrected *P*-distances are shown on the diagonal.

The newly discovered *Cyrtodactylus* population from Ban Thathom, Xiangkhoang Province, Laos, is genetically divergent from all other members of the *C. phongnhakebangensis* species group, and it is most closely related to *C. multiporus* (*P*=9.5%), *C. teyniei* (*P*=9.3%), and *C*. cf. *jarujini* (*P*=4.5%). The last population from Bolikhamxay Province of Laos was tentatively identified as *C*. cf. *jarujini* by Luu et al. (2016a); however, its taxonomic status requires further confirmation.

### Systematics

Based on the morphological, chromatical, and genetic distinctiveness of the newly discovered *Cyrtodactylus* population from all other populations in northern Laos and neighboring areas (see Diagnosis and Comparisons), and also on the results of phylogenetic analyses of the partial *COI* gene fragment, indicating that this population represents a clearly distinct mtDNA lineage, different from all congeners for which homologous sequences are available (see Results), we conclude that the Ban Thathom population represents a previously undescribed new species, which we describe and name herein.

### *Cyrtodactylus thathomensis* p. nov. (Figure 3, Figure 4, Figure 5 and Figure 6)

**Figure 3:**
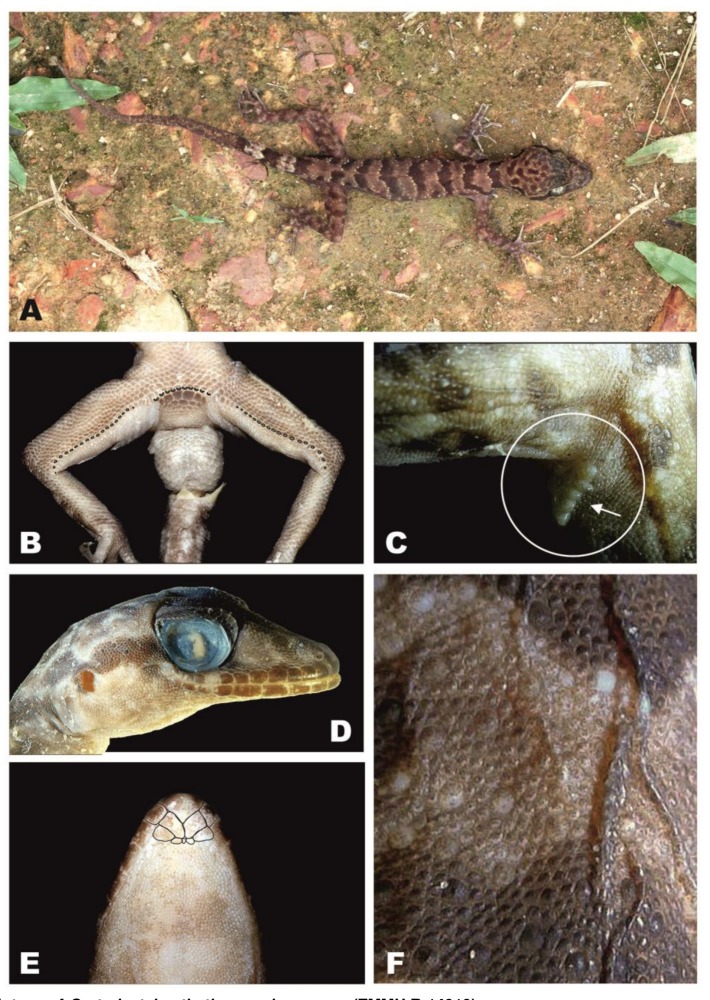
Male holotype of *Cyrtodactylus thathomensis* sp. nov. (ZMMU R-14919)

**Holotype**: ZMMU R-14919-1 (Figure 3); an adult male from the north-western slope of a limestone hill (N18°59′48.9″, E103°35′30.6″; alt. 271 m a.s.l.) near Ban (=Village) Thathom, Xiangkhoang Province, Laos. Collected on 14 May 2008 by E. L. Konstantinov and A. S. Chulisov.

A: General view of holotype in situ; B: Precloacal region with row of femoral and precloacal pores separated by diastema; C: Postcloacal spurs (see arrow); D: Lateral side of the head with supralabials and infralabials scalation; E: Ventral surface of the head with mental scalation; F: Dorsum surface with the rows of enlarged tubercles. Photos by E.L. Konstantinov (A) and R.A. Nazarov (B–F).

**Paratypes**: ZMMU R-15384 and ZISP 29731; two adult females from the same locality 

**Diagnosis**: *Cyrtodactylus thathomensis* **sp. nov.** can be distinguished from all other congeneric species by its medium body size (maximal known SVL 75.5 mm); dorsal tubercles in 14–18 rows at midbody; midbody scale rows 30–36 across belly between ventrolateral skin folds; a continuous series of 10–12 pore-bearing (male) or pitted (females), enlarged precloacal scales, separated by a diastema from a series of enlarged femoral scales bearing 18 or 19 pores (male) or 1–9 pits (females) on each femur; absence of precloacal groove; transversely enlarged median subcaudal scales; and four dark dorsal bands between limb insertions. 

**Description of holotype**: Adult male. SVL 70.3 mm. TailL 79.0 mm (last 54 mm regenerated). Head relatively long (HeadL 20.1; HeadL/SVL 0.29) and wide (HeadW 13.4; HeadW/HeadL 0.67), not markedly depressed (HeadH 8.4), distinct from neck. Loreal region inflated, canthus rostralis slightly prominent. Snout elongate (SnOrb/HeadL 0.41), rounded, longer than orbit diameter (OrbD/SnOrb 0.62). Scales on snout small, rounded to oval, granular to weakly conical, mostly homogeneous, larger than those on crown, interorbital, and occipital regions. Eye relatively large (OrbD/HeadL 0.25); pupil vertical with crenelated margins; supraciliaries short, larger anteriorly. Ear opening vertically oval, of moderate size (EarL/HeadL 0.06); orbit to ear distance subequal to orbit diameter (OrbEar/OrbD 1.02). Rostral much wider (3.5 mm) than deep (2.0 mm); rostral crease straight, starting from the upper middle of the rostral, going down half the rostral height. Two enlarged supranasals separated from one another by one internasal. Rostral contacting first supralabial on each side, nostrils, two supranasals and one internasal. Nostrils rounded, more or less laterally directed, each surrounded by supranasal, rostral, first supralabial and two postnasals. Three or four rows of small scales separate orbit from supralabials. Mental triangular, wider (3.4 mm) than deep (2.2 mm). A single pair of greatly enlarged postmentals in broad contact behind mental, each bordered anteromedially by mental, anterolaterally by first infralabial, posterolaterally by an enlarged lateral chinshield, and posteriorly by two granules (in total three granules contact the postmentals) (Figure 3E). Supralabials to mid-orbital position 9/9, enlarged supralabials to angle of jaws 10/11. Infralabials 9/9 (Figure 3D). Interorbital scale rows across narrowest point of frontal bone 30.

Body moderately slender, relatively short (AG/SVL 0.43) with poorly defined, non-denticulate, ventrolateral skin folds. Dorsal scales weakly heterogeneous, domed, with tubercles about four times size of adjacent dorsal scales extending from neck onto tail base, smaller tubercles on postocular region, crown and occiput; tubercles smooth or bearing a very small keel, tubercles on posterior trunk and sacral region most prominent. Dorsal tubercles in 18 rows at midbody, typically separated from one another by two dorsal granules (Figure 3F). Paravertebral tubercles 26. Ventral scales larger than dorsal scales, smooth, oval and subimbricate, largest in precloacal region. Midbody scale rows across belly between ventrolateral folds 30. Gular region with homogeneous, smooth, juxtaposed granular scales. A patch of enlarged precloacal scales on top of which lies a continuous series of ten pore-bearing scales, separated by a diastema of 3/4 enlarged poreless scales from a continuous series of 19/18 pore-bearing femoral scales. Enlarged femoral scales nearly two times the size of the scales of the adjacent anterior scale row (Figure 3B). No precloacal groove. Hemipeneal bulges evident. Postcloacal spurs bearing 6/5 enlarged conical scales (Figure 3C).

Scales on palm and sole smooth, rounded to oval or hexagonal, slightly domed. Scalation on dorsal surfaces of hind limbs similar to body dorsum with enlarged tubercles interspersed among smaller scales; tubercles smaller and rare on forelimbs. Forelimbs and hind limbs moderately long (ForeaL/SVL 0.16, TibiaL/SVL 0.19), moderately slender. Digits long, slender, inflected at interphalangeal joints, all bearing robust, slightly recurved claws. Basal subdigital lamellae broad, oval to rectangular, without scansorial surfaces; lamellae distal to digital inflection narrow; SLF4 17/17; SLT4 20/20. Subcaudals scales larger than supracaudal scales, forming a row of strongly enlarged transverse plates.

**Coloration in preservative**: Dorsal ground color of head, neck, body, limbs and tail light brown. Dorsal surface of head with irregular dark brown markings. Rostral, supralabials and infralabials dark brown, posterior ones heavily maculated with beige. On each side a postocular stripe reaching the nape but not meeting the one of the opposite side as it breaks into spots (i.e., a discontinuous nuchal collar). Upper surface of limbs showing irregular dark brown bands. Dorsum showing four dark brown bands between limb insertions. Each of the four bands on dorsum posteriorly limited by a discontinuous series of whitish tubercles, similarly to the band above shoulders, the one on the neck and the one above sacrum. Original part of the tail showing three dark brown bands. Regenerated part of the tail light brown. Undersurfaces of the head, throat, venter and members uniformly beige. Coloration of the holotype in life is shown in Figure 3A. In life its dorsal ground color is darker than in preservative. The tubercles posteriorly bordering the four bands on dorsum are lighter and more contrasting than after preservation. The yellow color of the outer extremities of the supraciliaries disappears in preservative.

**Figure 4:**
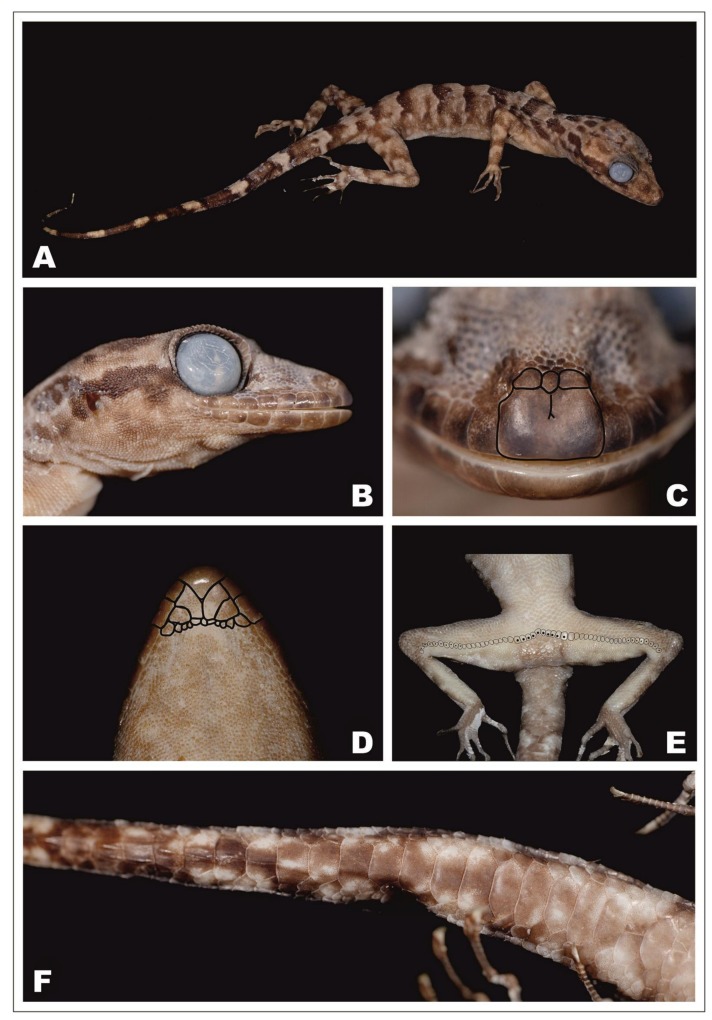
Female paratype of *Cyrtodactylus thathomensis* sp. nov. (ZISP 29731)

**Figure 5:**
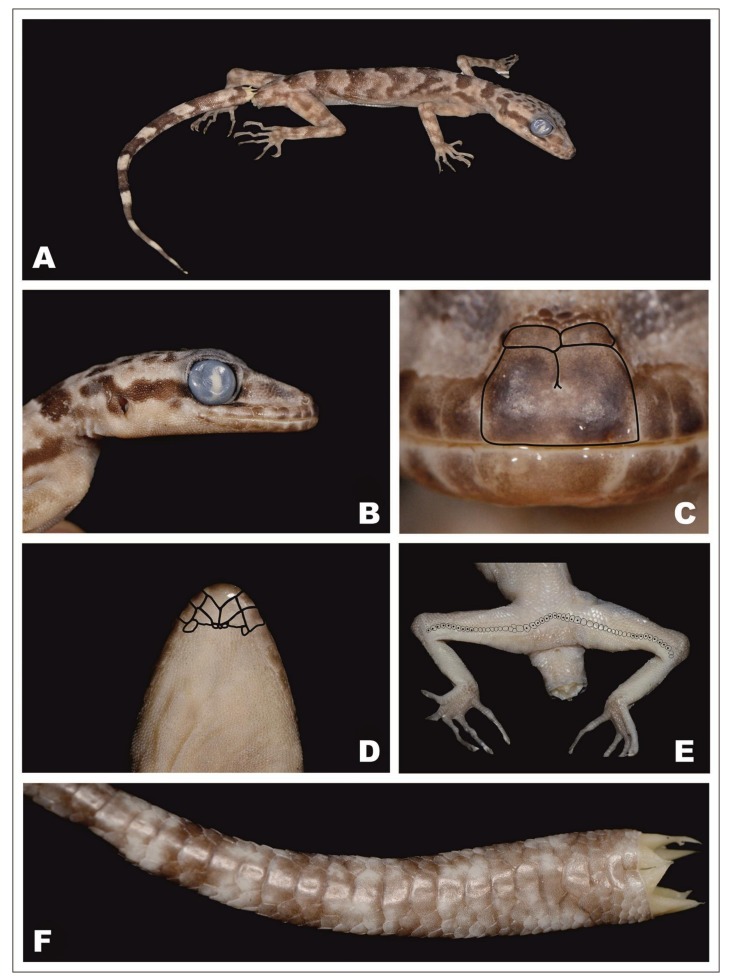
Female paratype of *Cyrtodactylus thathomensis* sp. nov. (ZMMU 15384)

**Figure 6:**
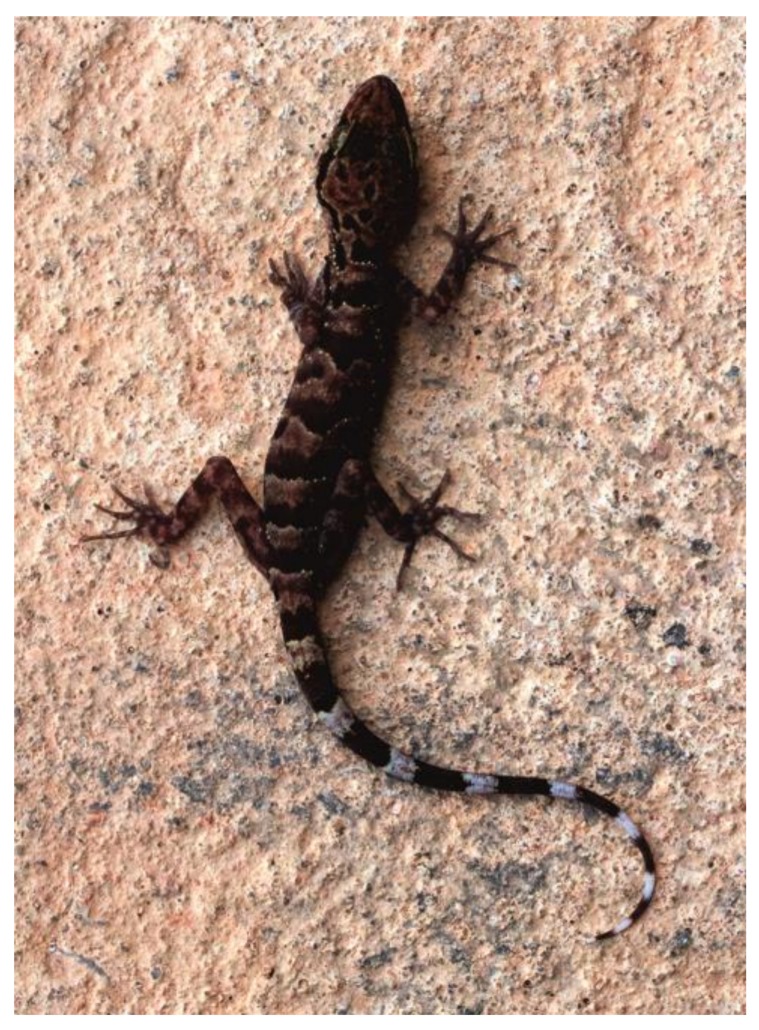
Live female paratype of *Cyrtodactylus thathomensis* sp. nov. (ZISP 29731) in dorsal view (Photo by E.L. Konstantinov)

**Variation:** Morphometric and meristic values for the type series are provided in Table 3. Morphological and coloration characters of the paratypes agree in most respects with those of the holotype, differing only in minor details (Table 3 and remarks hereafter). Rostral crease in paratypes like in holotype. One internasal in paratype ZISP 29731 like in holotype (Figure 4C), no internasal in paratype ZMMU R-15384 (hence supranasals in large contact) (Figure 5C). The female paratype ZISP 29731 shows a continuous row of 52 enlarged femoral-precloacal scales including, from left to right: 1 pitless scale + 9 scales with pits + 12 pitless scales + 10 precloacal scales with pits + 11 pitless scales + 9 scales with pits + 1 pitless scale (Figure 4E). The female paratype ZMMU R-15384 shows a continuous row of 55 enlarged femoral-precloacal scales including, from left to right: 10 scales with pits + 11 pitless scales + 12 enlarged precloacal scales with pits + 12 pitless scales + 8 scales with pits + 2 pitless scales (Figure 5E). General background dorsal color in preservative lighter in the female paratypes than in the male holotype. The paratypes’ tails, original and complete, show nine dark brown bands, and they are longer than SVL (TailL/SVL ratio 1.16–1.27; see Figure 4A– Figure 5A, Figure 6).

A: General view of preserved paratype with original tail; B: Lateral side of the head with supralabials and infralabials scalation; C: Shape of rostral and surrounding scales; D: Ventral surface of the head with mental scalation; E: Precloacal region with row of enlarged femoral and precloacal scales with pit and pitless scales; F: Medial row of enlarged subcaudal scales. Photos by R.A. Nazarov.

A: General view of preserved paratype with original tail; B: Lateral side of the head with supralabials and infralabials scalation; C: Shape and scalation of rostral (supranasals in contact with each other); D: Ventral surface of the head with mental scalation; E: Precloacal region with row of enlarged femoral and precloacal scales with pit and pitless scales; F: Medial row of enlarged subcaudal scales. Photos by R.A. Nazarov.

**Table 3:** Meristic and morphometric (in mm) data for the type series of *Cyrtodactylus thathomensis* sp. nov.

	ZMMU R-14919- 1Holotype	ZMMU R-15384 Paratype	ZISP 29731 Paratype
Sex	Male	Female	Female
SVL	70.3	71.7	75.5
ForeaL	11.4	12.0	12.7
TibiaL	13.7	12.8	12.8
TailL	79.0 (only first 25 mm original)	83.0	96.0
TailW	4.5	4.9	5.1
AG	30.5	31.2	31.7
HeadL	20.1	20.0	21.2
HeadW	13.4	12.8	14.3
HeadH	8.4	7.4	8.4
RW	3.5	2.8	2.8
RH	2.0	2.2	2.1
MW	3.4	3.2	3.1
ML	2.2	2.2	2.2
OrbD	5.1	4.8	5.0
OrbEar	5.2	5.0	5.7
SnOrb	8.2	7.8	8.2
NosOrb	6.2	6.0	6.5
Interorb	4.8	4.7	5.1
EarL	1.3	1.2	1.4
Internar	2.9	2.3	2.4
DorTub	18	18	14
ParaTub	26	32	24
PreclPi/PreclPo	10 Po	12 Pi	10 Pi
EnlFemSc	22/20	20/20	21/21
FemPi/FemPo	19 + 18 Po	10 + 8 Pi	9 + 9 Pi
Ven	30	32	36
SL	10/11	11/10	10/11
IL	9/9	9/10	10/10
InterorbSc	30	32	33
SLF4	17/17	17/16	16/16
SLT4	20/20	20/20	19/18

Paired meristic characters are given left/right.

**Comparison with congeneric species**: Synoptic tables comparing the main morphological characters of Lao *Cyrtodactylus* with the species known from adjacent regions were provided by Teynié & David (2014: 471–472) and Luu et al. (2016a, 2016b, 2016c). Among these species, we are comparing hereafter *Cyrtodactylus thathomensis* **sp. nov.** with all congeneric species found within a 500-km radius from its type-locality (a radius far superior to any maximal distance between two localities known for any species in the Indochinese Region, Thailand and Myanmar, cf. maps provided by Ellis & Pauwels, 2012; Nazarov et al., 2014: Figure 1; Grismer et al., 2015: Figure 1 – considering that *Cyrtodactylus intermedius* is a species complex composed of at least six species, *loc. cit*.: 114; Nguyen et al., 2014, 2017: Figure 1).

By its possession of transversely enlarged subcaudals, the new species is readily distinguished from *Cyrtodactylus angularis* (Smith, 1921), *C. buchardi* David, Teynié & Ohler, 2004, *C. cryptus* Heidrich, Rösler, Vu, Böhme & Ziegler, 2007, *C. jarujini*, *C. papilionoides* Ulber & Grossmann, 1991, *C. pseudoquadrivirgatus* Rösler, Nguyen, Vu, Ngo & Ziegler, 2008, and *C. vilaphongi* Schneider, Nguyen, Le, Nophaseud, Bonkowski & Ziegler, 2014. The possession of enlarged femoral scales separates *Cyrtodactylus thathomensis* **sp. nov.** from *C. bobrovi* Nguyen, Le, Pham, Ngo, Hoang, Pham & Ziegler, 2015, *C. buchardi*, *C. chauquangensis* Hoang, Orlov, Ananjeva, Johns, Hoang & Dau, 2007, *C. cryptus*, *C. otai* Nguyen, Le, Pham, Ngo, Hoang, Pham & Ziegler, 2015, *C. pageli*, *C. pseudoquadrivirgatus*, *C. spelaeus* Nazarov, Poyarkov, Orlov, Nguyen, Milto, Martynov, Konstantinov & Chulisov, 2014, *C. vilaphongi* and *C. wayakonei* Nguyen, Kingsada, Rösler, Auer & Ziegler, 2010.

The presence of precloacal and femoral pores separated by a diastema in male *Cyrtodactylus thathomensis* **sp. nov.** distinguishes this species from *C. angularis*, *C. bansocensis*, *C. bobrovi*, *C. buchardi*, *C. calamei*, *C. chanhomeae* Bauer, Sumontha & Pauwels, 2003, *C. chauquangensis*, *C. cryptus*, *C. cucphuongensis* Ngo & Chan, 2011, *C. darevskii*, *C. hinnamnoensis*, *C. jaegeri*, *C. jarujini*, *C. khammouanensis*, *C. lomyenensis*, *C. martini* Ngo, 2011, *C. multiporus*, *C. otai*, *C. pageli*, *C. papilionoides*, *C. phongnhakebangensis*, *C. pseudoquadrivirgatus*, *C. puhuensis* Nguyen, Yang, Le, Nguyen, Orlov, Hoang, Nguyen, Jin, Rao, Hoang, Che, Murphy & Zhang, 2014, *C. roesleri*, *C. rufford*, *C. sommerladi*, *C. soudthichaki*, *C. spelaeus*, *C. vilaphongi* and *C. wayakonei*. *Cyrtodactylus teyniei* was described based on a single female holotype. Teynié & David (2014) reported the first known male; it showed precloacal and femoral pores, but they did not mention if the femoral and precloacal pored scales were separated by a diastema or not. It should be noted that, while in the male holotype of *Cyrtodactylus bansocensis* the precloacal and femoral pore-bearing scales are separated by a diastema, they are not in the male paratype (Luu et al., 2016c).

Its banded dorsal pattern separates *Cyrtodactylus thathomensis* **sp. nov.** from *C. buchardi*, *C. jarujini*, *C. multiporus*, *C. pseudoquadrivirgatus*, *C. spelaeus* and *C. teyniei*, which show blotched patterns. Additional differences distinguishing the new species from *Cyrtodactylus jarujini* include its smaller SVL (75.5 vs. 90 mm in *C. jarujini*) and a lower total number of pores in males (47 vs. 52–54). Additional differences with *Cyrtodactylus multiporus* include the new species’ much smaller SVL (75.5 vs. 98 mm in *C. multiporus*) and a much lower total number of pores in males (47 vs. 58–60). From *Cyrtodactylus teyniei* the new species differs also by its smaller SVL (75.5 vs. 89.9 mm in *C. teyniei*), generally lower number of midbody scale rows across belly between ventrolateral folds (30–36 vs. 36–38), and a much lower total number of pores in males (47 vs. 58).

Among the remaining species living within the 500-km radius of its type-locality, *Cyrtodactylus thathomensis* sp. nov. can be differentiated from *C. auribalteatus* Sumontha, Panitvong & Deein, 2010 by its much smaller SVL (75.5 vs. 98.1 mm), lower DorTub (14–18 vs. 22–24), lower Ven (30–36 vs. 38–40), and higher FemPo on each side (18 or 19 vs. 4 or 5) and PreclPo (10 vs. 6) in males; from *C. bichnganae* Ngo & Grismer, 2010 by its much smaller SVL (75.5 vs. 99.9 mm), much higher EnlFemSc on each side (20–22 vs. 11–13), and much higher FemPo on each side in males (18 or 19 vs. 9); from *C. doisuthep* Kunya, Panmongkol, Pauwels, Sumontha, Meewasana, Bunkhwamdi & Dangsri, 2014 by its smaller SVL (75.5 vs. 90.5 mm), lower DorTub (14–18 vs. 19 or 20), presence of femoral pores (vs. pits) in males, higher PreclPo (10 vs. 6), and 4 (vs. 5 or 6) dark bands between limb insertions; from *C. dumnui*i Bauer, Kunya, Sumontha, Niyomwan, Pauwels, Chanhome & Kunya, 2010 by its generally lower DorTub (14–18 vs. 18–22), its lower Ven (30–36 vs. 40), and much higher FemPo on each side (18 or 19 vs. 6 or 7) and PreclPo (10 vs. 5 or 6) in males; from *C. huongsonensis* Luu, Nguyen, Do & Ziegler, 2011 by its by its smaller SVL (75.5 vs. 89.8 mm), lower Ven (30–36 vs. 41–48), higher PreclPo (10 vs. 6) and much higher FemPo on each side (18 or 19 vs. 7–10) in males, and its discontinuous (vs. continuous) nuchal loop; from *C. interdigitalis* Ulber, 1993 by its generally lower DorTub (14–18 vs. 18–22), its lower Ven (30–36 vs. 37–42), much higher FemPo on each side (18 or 19 vs. 9) and lower PreclPo (10 vs. 14) in males, and absence (vs. presence) of webbing; from *C. intermedius* ( Smith, 1917) by its lower Ven (30–36 vs. 40–50) and much higher EnlFemSc on each side (20–22 vs. 6–10); from *C. inthanon* Kunya, Sumontha, Panitvong, Dongkumfu, Sirisamphan & Pauwels, 2015 by its generally lower DorTub (14–18 vs. 18–20), and much higher FemPo on each side (18 or 19 vs. 6) and PreclPo (10 vs. 5) in males; from *C. khelangensis* Pauwels, Sumontha, Panitvong & Varaguttanonda, 2014 by its much smaller SVL (75.5 vs. 95.3 mm), and much higher FemPo on each side (18 or 19 Po vs. 6 or 7 Po or Pi) and PreclPo (10 vs. 2–5) in males; from *C. kunyai Pauwels*, Sumontha, Keeratikiat & Phanamphon, 2014 by its much higher FemPo on each side (18 or 19 vs. 5 or 6) and PreclPo (10 vs. 3) in males; and from *C. soni Le*, Nguyen, Le & Ziegler, 2016 by its much smaller SVL (75.5 vs. 103.0 mm), higher DorTub (14–18 vs. 10–13), lower Ven (30–36 vs. 41–45), and much higher FemPo on each side (18 or 19 vs. 6–8) and PreclPo (10 vs. 6 or 7) in males.

Besides differentiating *Cyrtodactylus thathomensis* **sp. nov.** from all congeneric species found within a 500-km radius, its combination of characters presented in the Diagnosis allows to unambiguously separate it from all species found in Bangladesh, Cambodia, Myanmar, Thailand and Vietnam (see, among other references in the literature cited, Bauer, 2003; Connette et al., 2017; Grismer et al., 2012; Le et al., 2016; Mahony et al., 2009; Mahony, 2009; Panitvong et al., 2014; Pauwels & Sumontha, 2014; Pauwels et al., 2014, 2016; Sumontha et al., 2014, 2015). 

**Figure 7:**
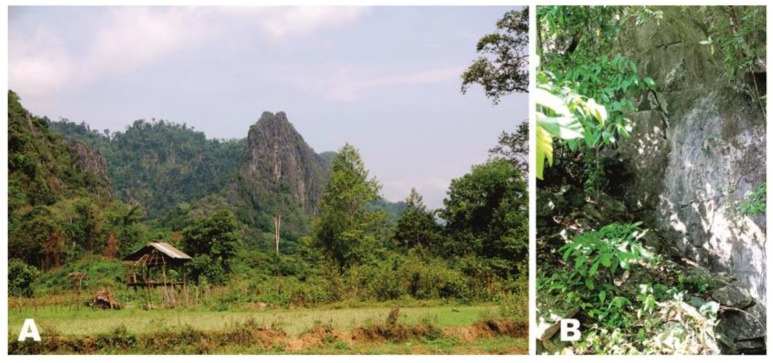
Habitat of *Cyrtodactylus thathomensis* sp. nov. at the type-locality

**Distribution and natural history**: *Cyrtodactylus thathomensis* **sp. nov.** is so far known only from its type-locality. The types were collected on karst boulders on a steep forested limestone hill (Figure 7).

A: General view of karst massif at Ban Thathom in Xiangkhoang Province, northeastern Laos; B: Microhabitat of *Cyrtodactylus thathomensis* **sp. nov.**. Photos by E.L. Konstantinov.

**Phylogenetic position**: The new species is a member of the *C. phongnhakebangensis* species group *sensu* Luu et al. (2016a) within which it is most closely related to *C. multiporus*, *C. teyniei* and *C*. cf. *jarujini* (see Results). 

**Etymology**: The specific epithet “*thathomensis*” is a Latinized toponymic adjective, referring to the type locality of the new species, Ban Thathom. We suggest the following common names: *Ki Chiem Thathom* (Lao), *Tuk Khai Thathom* (Thai), *Thathom Bent-toed Gecko* (English), *Tatomskiy Krivopalyi Gekkon* (Russian), *Thathomkromvingergekko* (Dutch), and *Cyrtodactyle de Thathom* (French).

## DISCUSSION

Mitochondrial genealogy indicates *Cyrtodactylus thathomensis* **sp. nov.** as a member of the *C. phongnhakebangensis* species group, within which it appears to be most closely related to *C. multiporus* and *C. teyniei*. Table 4 compares the main diagnostic characters of the species included in the *C. phongnhakebangensis* species group *sensu* Luu et al. (2016a) and our new species. All species in this group, including the one newly described here, live in the Cammon Plateau and the southern edge of the Xiangkhoang Plateau in the Annam Cordillera. This species group currently represents 15 out of the 22 *Cyrtodactylus* species presently recorded from Laos (i.e., 68%), with the remaining seven species belonging to the *C. interdigitalis*, *C. irregularis* and *C. wayakone*i species groups *sensu* Luu et al. (2016a).

**Table 4:** Main diagnostic characters of species included in the *Cyrtodactylus phongnhakebangensis* species group *sensu* Luu et al. (2016a)

Species	Maximum SVL (mm)	Banded (Ba) / blotched (Bl) pattern	Dorsum dark bands straight(S) / curved (C)	Nuchal collar continuous (C) / discontinuous (D)	Number of dark bands between limb insertions	Number of dark bands on tail	DorTub	Ven	FemPreclPo (males)	PreclPo (males)	FemPo (males)
*C. thathomensis* **sp. nov.**	75.5	Ba	C	D	4	9	14–18	30–36	–	10	18–19 (One side)
*C. bansocensis*	74.0	Ba	**S**	**C**	**3**	**11**	14–15	34–35	1	1	1
*C. calamei*	**89.3**	Ba	C	**C**	**3**	>8	10–16	**39–42**	**35–39**	–	–
*C. darevskii*	**100.0**	Ba	C	**C**	**3**	8–10	16–20	**38–46**	**38–44**	–	–
*C. hinnamnoensis*	**100.6**	Ba	C	**C**	**3**	>8	13–19	35–48	**36–44**	–	–
*C. jaegeri*	68.5	Ba	**S**	**C**	**3**	**>15**	15–17	31–32	**44**	–	–
*C. jarujini*	**90.0**	**Bl**	–	D	–	?	18–20	32–38	**52–54**^**2**^	–	–
*C. khammouanensis*	73.0	Ba	**S**	**C**	**3**	>2	16–21	32–38	**40–44**	–	–
*C. lomyenensis*	71.2	Ba	**S**	**C**	**3**	**12**	**20–24**	35–36	**39–40**	–	–
*C. multiporus*	**98.0**	**Bl**	–	D	–	**6–8**	16–20	30–38	**58–60**	–	–
*C. pageli*	81.8	Ba	C	**C**	4–5	8–10	9–14	**41–46**	–	**4**	**0**
*C. phongnhakebangensis*	**96.3**	Ba	C	**C**	**2–3**	**16**^**3**^	11–20	32–42	**32–42**	–	–
*C. roesleri*	75.3	Ba	C	**C**	**3**	**10**	13–19	34–40	**20–28**	–	–
*C. rufford*	72.5	Ba	**S**	**C**	**3**	>7	14–16	**27–29**	**42–43**	–	–
*C. sommerladi*	80.3	Ba	C	**C**^**4**^	3–4	**12**	**0**	31–39	**20–26**	–	–
*C. soudthichaki*	70.0	Ba	C	**C**^**5**^	**3**	**15**	**19–20**	32–33	**29**	–	–
*C. teyniei*	**89.9**	**Bl**	–	D	–	**12**	**19**	36–38	586	?	?

^1^: 34 FemPreclPo in paratype and holotype, but respectively continuous and interrupted; ^2^: Partly interrupted as described by Ulber (1993); ^3^: From Luu et al. (2016a: Figure 9A); ^4^: Interrupted in only one of the 15 specimens of the type series (see original description by Luu et al., 2016a); ^5^: Briefly interrupted on the right side of the holotype; ^6^: It cannot be clearly deduced from Teynié & David (2014) if the pored precloacal and femoral scales are in a continuous series or not. Characters in bold indicate a diagnostic difference with the new species.

As far as we know, *Cyrtodactylus thathomensis* **sp. nov.** is not found in the pet trade nor used in traditional medicine. The type-locality not being located within a protected area, the main potential threats to this new gecko species are habitat destruction through deforestation and limestone exploitation. To date, however, there appears to be no immediate concern as to the conservation status of this species, despite its limited distribution. Our new discovery stresses again the necessity to systematically survey karst massifs to inventory their unique, often micro-endemic and fragile, biodiversity.

## APPENDIX I

List of examined specimens (Distr.=District, Prov.=Province).

*Cyrtodactylus bidoupimontis*: ZMMU R-13368 (holotype), northern slope of Bidoup Mountain, near Klong Klanh village, Bidoup - Nui Ba National Park, Da Chais commune, Lac Duong Distr., Lam Dong Prov., Vietnam; ZMMU R-13369-1–5 (paratypes), along the road to Nha Trang, 6–7 km from Klong Klanh, Da Nhim river valley in Bidoup - Nui Ba National Park, Lam Dong Prov., Vietnam; additional specimens listed in Nazarov et al. (2012).

*Cyrtodactylus brevipalmatus*: THNHM 3121–3123, THNHM 3125, Khao Phanoen Thung Camp, Kaeng Krachan National Park, Kaeng Krachan Distr., Phetchaburi Prov., Thailand.

*Cyrtodactylus bugiamapensis*: ZISP 26323 (paratype), ZMMU R-13366 (holotype), ZMMU R-13367-1–4 (paratypes), Bu Gia Map National Park, Dak Ka stream valley, Bu Gia Map commune, Bu Gia Map Distr., Binh Phuoc Prov., Vietnam; additional specimens listed in Nazarov et al. (2012).

*Cyrtodactylus chanhomeae*: CUMZ R 2003.62 (paratype), Thep Nimit Cave, Khun Khlon Subdistrict, Phraputthabata Distr., Saraburi Prov., Thailand; IRSNB 2585 (holotype), Phraya Chat-tan Cave, Khun Khlon Subdistrict, Phraputthabata Distr., Saraburi Prov., Thailand.

*Cyrtodactylus doisuthe*p: CUMZ R 0.2318 (paratype), QSMI 1168 (paratype), THNHM 22543 (holotype), Doi Suthep, Muang Distr., Chiang Mai Prov., Thailand.

*Cyrtodactylus dumnuii*: CUMZ R 2009-6-24-5–6 (paratypes), KZM 002 (paratype), THNHM 15904 (holotype), THNHM 15905 (paratype), Tham (Cave) Phabartmaejon, Ban Thakilek, Mae-Na Subdistrict, Chiang Dao Distr., Chiang Mai Prov., Thailand.

*Cyrtodactylus erythrops*: THNHM 15377 (holotype), Tham Lod, Pangmapha Distr., Mae Hong Son Prov., Thailand.

*Cyrtodactylus intermedius*: IRSNB 17011, Nakhon Ratchasima, Thailand; ZMMU R- 11213-1, Phnom Bakor National Park, Cambodia.

*Cyrtodactylus inthanon*: CUMZ R 0.2320 (paratype), THNHM 22550 (holotype), THNHM 25605 (paratype), Doi Inthanon, Jom Thong Distr., Chiang Mai Prov., Thailand.

*Cyrtodactylus khelangensis*: CUMZ R 0.2318 (paratype), PSUZC-RT 2012.9 (paratype), THNHM 22548 (holotype), limestone cave in Pratu Pha, Mae Mo Distr., Lampang Prov., Thailand.

*Cyrtodactylus kunyai*: THNHM 2560 (holotype), limestone cave in Suan Hin Pha Ngam, Nong Hin Distr., Loei Prov., Thailand.

*Cyrtodactylus lekagul*i: IRSNB 2678 (paratype), MNHN 1998.0600, Tham Phung Chang, Phang-Nga City, behind Provincial Hall, Muang Distr., Phang-Nga Prov., Thailand; MNHN 1999.7706, Tham Reusi, Muang Distr., Phang-Nga Prov., Thailand. *Cyrtodactylus* cf. lekaguli: IRSNB 16558, Nakhon Si Thammarat Prov., Thailand.

*Cyrtodactylus oldhami*: MNHN 1999.7626, MNHN 1999.7698, Phang-Nga Wildlife Breeding Station, Muang Distr., Phang-Nga Prov., Thailand.

*Cyrtodactylus peguensis peguensis*: MNHN 1998.0544, Ban Kok Jaroen, Tap-Phut Distr., Phang-Nga Prov., Thailand.

*Cyrtodactylus phetchaburiensis*: IRSNB 2682 (holotype), Tham Khao Tomo, Khao Tomo, Klatluang Subdistrict, Tha Yang Distr., Phetchaburi Prov., Thailand; IRSNB 2683 (paratype), Phetchaburi Prov., Thailand.

*Cyrtodactylus phuketensis*: PSUZC-RT 2010.58 (holotype), QSMI 1170 (paratype), THNHM 15378 (paratype), Ban Bangrong, Thalang Distr., Phuket Island, Phuket Prov., Thailand.

*Cyrtodactylus ranongensis*: PSUZC-RT 2012.7–8 (paratypes), THNHM 22545 (holotype), Ban Ton Kloy, Kampuan Subdistrict, Suk Samran Distr., Ranong Prov., Thailand; THNHM 22546 (paratype), along a street near Phetchkasem Road, Ban Kampuan, Kampuan Subdistrict, Suk Samran Distr., Ranong Prov., Thailand.

*Cyrtodactylus saiyok*: THNHM 25602 (holotype), CUMZ R 0.2320 (paratype), Moo 3, Wang Krajae Subdistrict, Sai Yok Distr., Kanchanaburi Prov., Thailand.

*Cyrtodactylus samroiyot*: CUMZ R 0.2320 (paratype), QSMI 1167 (paratype), THNHM 22549 (holotype), Ban Bang Pu, Sam Roi Yot Distr., Prachuap Khiri Khan Prov., Thailand.

*Cyrtodactylus sanook*: QSMI 1165 (paratype), PSUZC-RT 2012.4 (paratype), THNHM 22541 (holotype), Wat Tham Sanook, Banna Subdistrict, Muang Distr., Chumphon Prov., Thailand.

*Cyrtodactylus sumonthai*: CAS 223819 (paratype), CUMZ R 2002.2.5.1 (paratype), IRSNB 2625 (paratype), Khao Wong, Rayong Prov., Thailand; IRSNB 2626 (paratype), Tham Tao, Khao Wong, Rayong Prov., Thailand; IRSNB 2624 (holotype), Tham Khang Khao, Khao Wong, Rayong Prov., Thailand.

*Cyrtodactylus thirakhupti*: CUMZ R 2003.120 (holotype), CUMZ R 2003.121 (paratype), IRSNB 2590 (paratype), Tham Khao Sonk, Thachana Distr., Surat Thani Prov., Thailand.

*Cyrtodactylus tigroides*: CUMZ R 2002.296C (holotype), IRSNB 2586 (paratype), Ban Tha Sao, Sai Yok Distr., Kanchanaburi Prov., Thailand.

*Cyrtodactylus wangkulangkulae*: THNHM 22547, limestone cave near Wangsaithong Waterfall, Manang Distr., Satun Prov., Thailand.

## APPENDIX II

General distribution of the genus *Cyrtodactylus* in Laos and surrounding areas, legend to Figure 1 (Prov.= Province).

Localities: 1 – *Cyrtodactylus* cf. *martini* – Xishuangbanna, Yunnan Prov., China; 2 – *C. martini* – Lai Chau, Lai Chau Prov., Vietnam (type locality); 3 – *C. bichnganae* – Son La, Son La Prov., Vietnam (type locality); 4 – *C. wayakonei* – Kao Rao Cave, Ban Nam Eng, Vieng Phoukha, Luang Nam Tha Prov., Laos (type locality); 5 – *C. ota*i – Hang Kia, Hang Kia – Pa Co N.R., Mai Chau District, Hoa Binh Prov., Vietnam (type locality); 6 – *C. bobrovi* – Hau 3, Ngoc Lau Commune, Ngoc Son – Ngo Luong N.R., Lac Son District, Hoa Binh Prov., Vietnam (type locality); 7 – *C. huongsonensis* – Huong Son, My Duc, Hanoi, Vietnam (type locality); 8 – *C. cucphuongensis* – Cuc Phuong N.P., Nho Quan, Ninh Binh Prov., Vietnam (type locality); 9 – *C. soni* – Da Han, Van Long Wetland N.R., Gia Vien District, Ninh Binh Prov., Vietnam (type locality); 10 – *C. puhuensis* – Pu Hu, Thanh Hoa Prov., Vietnam (type locality); 11 – *C*. cf. *puhuensis* – Houphan Prov., Laos; 12 – *C. vilaphongi* – Ban Xieng Muak, Luang Prabang, Luang Prabang Prov., Laos (type locality); 13 – *C. chauquangensis* – Chau Quang, Quy Hop, Nghe An Prov., Vietnam (type locality); 14 – *C*. cf. *interdigitalis* and *C. spelaeus* – Khuang Lang N.P., Kasi, Vientiane Prov., Laos (type locality of *C. spelaeus*); 15 – *C. pageli* – Phoukham Cave, Ban Na Thong, Vang Vieng, Vientiane Prov., Laos (type locality); 16 – *Cyrtodactylus thathomensis* sp. nov. – Ban Thathom, Xiangkhouang Prov., Laos (type locality); 17 – *C*. cf. *jarujini* – Phou Khao Khouay N.P., Bolikhamxay Prov., Laos; 18 – *C. interdigitalis* – Nam Lik River Valley, Ban That Wang Monh, Vientiane Prov., Laos; 19 – *C. teyniei* – Ban Na Hin (Nahin), Nam Kading NBCA, Bolikhamxay Prov., Laos (type locality); 20 – *C. jarujini* – Phu Wua W.S., Nong Dern, Bung Kan, Nong Khai Prov., Thailand (type locality); 21 – *C*. cf. *teyniei* – Nahin, Khammouane Prov., Laos; 22 – *C*. cf. *roesleri* – Phou Hin Boun N.P., Konglor, Khammouane Prov., Laos; 23 – *C. soudthichaki* – Khun Don, Phou Hin Poun N.P., Khammouane Prov., Laos (type locality); 24 – *C. jaegeri* – Thakhek, Khammouane Prov., Laos (type locality); 25 – *C. lomyenensis* – Lomyen Cave, Gnommalath, Khammouane Prov., Laos (type locality); 26 – *C. rufford* – Nang Log cave, Gnommalath District, Khammouane Prov., Laos (type locality); 27 – *C. darevskii*, *C. khammouanensis* and *C. multiporus* – Na Home, Boulapha, Khammouane Prov., Laos (type locality); 28 – *C. bansocensis* – Peopalam Cave, Ban Soc, Bualapha District, Khammouane Prov., Laos (type locality); 29 – *C. calamei*, *C. hinnamnoensis*, *C. sommerladi*, *C. cryptus* – Hin Nam No N.P., Khammouane Prov., Laos (type locality for *C. calamei*, *C. hinnamnoensis* and *C. sommerladi*); 30 – *C. roesleri* – Ke Go N.R., Ha Tinh - Quang Binh provincial border, Vietnam; 31 – *C. phongnhakebangensis*, *C. cryptus* and *C. roesleri* – Phong Nha – Ke Bang N.P., Minh Hoa, Quang Binh Prov., Vietnam (type locality); 32 – *C. kunyai* – Suan Hin Pha Ngam, Nong Hin District, Loei Prov., Thailand (type locality); 33 – *C. interdigitalis* – Tham Yai Nam Nao, Nam Nao N.P., Phetchabun Prov., Thailand (type locality); 34 – *C. pseudoquadrivirgatus* – Huong Hoa, Quang Tri Prov., Vietnam; 35 – *C. pseudoquadrivirgatus* – A Luoi, Thua Thien – Hue Prov., Vietnam (type locality); 36 – *Cyrtodactylus* sp. 1 – Ba Na, Da Nang, Vietnam; 37 – *C. buchardi* – Kiatngong, Xepian NBCA, Champasak Prov., Laos; 38 – *C. taynguyenensis* – Kon Plong, Kon Tum Prov., Vietnam; 39 – *C. taynguyenensis* – Krong Pa (type locality), Kon Ka Kinh N.P. and Kon Chu Rang N.R., Gia Lai Prov., Vietnam.
